# HIV-Infected Late Presenter Patients

**DOI:** 10.1155/2012/902679

**Published:** 2011-11-29

**Authors:** Antonella D'Arminio Monforte, Andrea Antinori, Enrico Girardi, Francesca Ceccherini-Silberstein, Giulia Marchetti, Caroline Anne Sabin, Julio S. Montaner

**Affiliations:** ^1^Department of Medicine, Surgery and Dentistry University of Milan Clinic of Infectious Diseases, “San Paolo” Hospital, Milan, Italy; ^2^National Institute for Infectious Diseases Lazzaro Spallanzani, via Portuense, Roma, Italy; ^3^Dipartimento di Epidemiologia, Istituto Nazionale Per le Malattie Infettive, Rome, Italy; ^4^Department of Experimental Medicine and Biochemical Sciences University of Rome “Tor Vergata” via Montpellier 1, 00133 Rome, Italy; ^5^Research Department of Infection and Population Health, UCL Medical School, Royal Free Campus, Rowland Hill Street, London NW3 2PF, UK; ^6^British Columbia Centre for Excellence in HIV/AIDS Research, University of British Columbia, Vancouver, BC, Canada; ^7^Faculty of Medicine, Vancouver, BC, Canada

The idea of dedicating a special issue of the journal to explore the challenges posed by late presentation of HIV infection is based upon several considerations. In 2010, after three decades since the beginning of the AIDS epidemics, still 15–38% of patients with HIV infection continue to be diagnosed late. As expected, late presenters demonstrate a less favourable clinical course, with reduced or incomplete treatment response, more rapid clinical progression, and higher risk of mortality. In addition, they generate a considerable and avoidable resource burden to the healthcare system. Furthermore, undiagnosed HIV-infected individuals contribute disproportionatelly to the spread of HIV disease. The latter has become even more important now that antiretroviral therapy has been conclusively shown to decrease HIV transmision by greater than 90%. As a result, expanding and even normalizing HIV testing is increassingly recognized by international guidelines to be a key public health priority, in order to improve access to care, to allow an earlier antiretroviral treatment initiation, and ultimately to decrease HIV-related morbidity and mortality as well as HIV transmission. 

This issue contains five key papers. L. Saganic and colleagues report a persistent unacceptably high proportion of late diagnoses of HIV in Washington state between 2000 and 2009, despite the implementation of CDC recommendations for HIV testing in the last five years. The conclusion is that HIV screening procedures, largely dependent on an individual's perception of his/her HIV risk, have failed; in fact, people who are more likely to be diagnosed late with HIV usually do not consider themselves as “risk categories” and have poor access to HIV testing. Consistent with several other publications, also in this study, heterosexuals, women, elderly people, foreign-born individuals, and people residing in a rural area showed higher risk of late HIV diagnosis. Interestingly, two different measures of late presentation are compared: the lab-based measure (CD4+ T-cell count at presentation <350 cells/mmc) and the time-based one (AIDS event within 12 months of initial HIV diagnosis). Both measures underline the same risk factors for late HIV testing. The laboratory-based definition, however, appears to be the more clinically relevant one due to the fact that it reflects the importance of the CD4+ T-cell count for determining the optimal time to initiated antiretroviral therapy; although the application of this definition may be limited in some settings, this should improve as the quality of laboratory systems improves in the future. Nevertheless, the contemporary use of both measures may offer a consistent assessment of the problem of late presentation.

Also in the study conducted by K. Buchacz and colleagues, the proportion of HIV-positive patients who first present a CD4+ T-cell count <350 cells/mmc at first presentation to care was extremely high (58%) consistent with the previous paper. K. Buchacz analyzed data from participants in the HIV Outpatient Study (HOPS) from eight USA cities, including Washington, over the same time period considered by L. Saganic (2000–2009). Again, similar risk factors for Late Presentation were identified (heterosexual transmission, older age, and nonwhite ethnicity). Interestingly, the median CD4+ T-cell count at HIV diagnosis remained stable over time, with only a trend towards higher CD4+ T-cell counts in those infected via heterosexual sex and people attending public facilities. Taken together, these studies provide a strong rationale for the normalization of HIV testing. 

One of the limitations of many analyses of late presentation is that definitions do not generally incorporate information on duration of infection prior to diagnosis (which is often unknown). The CD4+ T-cell count at diagnosis is therefore used as a surrogate for this. To get around this problem, P. Sobrino-Vegas et al. used a multiple imputation method to estimate the probable date of HIV seroconversion for all patients in their Spanish cohort (some of whom were known seroconverters); late diagnosis was then defined as an interval of greater than 4.19 years between the time of seroconversion and the time of first testing. This group, which comprised 34% of the cohort, were more often male, older, and of non-Spanish origin. Using this definition, 39% of newly diagnosed subjects had presented late; these individuals were more commonly intravenous drug users. Use of this different approach to the estimation of late diagnosis underlines the importance of further strengthening HIV screening procedures.

From an immunological perspective, F. Bai et al. reported that late presenters tend to be characterized by CD127 down-regulation on CD4+ T-cells and immune activation; as these patients are in an advanced stage of infection and are at higher risk of disease progression and of poor immune reconstitution, when they start antiretroviral therapy, peripheral T lymphocytes immune phenotypes could be proposed as adjunctive markers to complement CD4+ count when attempting to identify and monitor late presenters. The distinctive immunological patterns seen in late presenters were similar regardless of the definition of late HIV diagnosis that was used: CD4+ T-cell count <350 cell/mmc (late presentation), CD4+ T-cell count <200 cells/mmc (Advanced HIV disease), or AIDS defining condition at presentation regardless of the patient's CD4+ T-cell counts (AIDS presentation). 

Finally, H. B. Krentz and M. J. Gill report a similarly high proportion of new patients who present late (59%) and describe the significantly higher costs incurred by this group, most notably when considering inpatient costs and any costs incurred during the first year after entry to care. In particular, the economic burden associated with late presentation remained elevated after the first year, at twice that of patients presenting with a CD4+ T-cell count >350 cells/mm^3^.

As underlined by these works, late presentation for HIV remains a key unresolved challenge in HIV/AIDS with serious adverse consequences at the individual and societal levels. The tools are available to fully address this problem. Implementation science and operations research initiatives are urgently needed to better define the best strategies to eliminate late presentation. This will be an essential step to control HIV morbidity, mortality, and transmission. 

*Antonella D'Arminio Monforte* *Andrea Antinori* *Enrico Girardi* *Francesca Ceccherini-Silberstein* *Giulia Marchetti* *Caroline Anne Sabin* *Julio S. Montaner*

